# Correction osteotomy for bilateral varus knee deformity caused by premature epiphyseal closure induced by hypervitaminosis A: a case report

**DOI:** 10.1186/s12891-019-2665-2

**Published:** 2019-06-15

**Authors:** Masatake Matsuoka, Tomohiro Onodera, Tokifumi Majima, Koji Iwasaki, Daisuke Takahashi, Eiji Kondo, Norimasa Iwasaki

**Affiliations:** 1 Department of Orthopaedic Surgery, Hokkaido University Graduate School of Medicine, North 15 West 7, Kita-Ku, Sapporo, 060-8638 Japan; 2 Department of Orthopaedic Surgery, Nippon Medical School, 1-1-5, Senndagi, Bunkyo-ku, Tokyo, 113-8603 Japan

**Keywords:** Retinoid therapy, Premature epiphyseal closure, 13-cis-retinoic acid, Osteopenia

## Abstract

**Background:**

A vitamin A derivative, 13-cis-retinoic acid (isotretinoin), has been administered to treat several types of pediatric cancer and has improved survival rates in patients despite being known to induce premature epiphyseal closure. As the number of patients treated by 13-cis-retinoic acid increases, demands for salvage treatment after systemic retinoid therapy are emerging. However, few studies have described the surgical treatment of this disease.

**Case presentation:**

We report a case with bilateral varus knee deformity due to premature epiphyseal closure that occurred during treatment with isotretinoin for neuroblastoma. The patient was successfully treated with correction osteotomy using a Taylor spatial frame in the right knee joint and femoral closed wedge osteotomy using a locking plate in left knee joint. Histopathological examination of the growth plate showed polar irregularity of chondrocytes and decreased cartilage matrix without apoptosis. In contrast, arthroscopic findings showed an intact joint surface. No recurrence of varus deformity was evident on follow-up at 1 year.

**Conclusions:**

To the best of our knowledge, this represents the first report of correction osteotomy for varus knee deformity due to premature epiphyseal closure that occurred during treatment with isotretinoin.

## Background

The first description of poisoning by vitamin A was described in 1596 and again in 1853 by arctic explorers who became ill after ingesting polar bear liver that had a high content of vitamin A [1, 2]. In the 1940s, Caffey described overdoses of vitamin A in seven children who presented with skeletal deformities including pain, swollen limbs and radiographic changes [3]. They had been erroneously taking excessive amounts of vitamin concentrate A and D over long periods as oral supplement given them by their parents.

A vitamin A derivative, 13-cis-retinoic acid (isotretinoin), offers a new potential treatment, as the modulation of endogenous retinoids induces cytodifferentiation and apoptosis [4]. This agent has thus been administered to treat several types of pediatric cancer and has improved survival rates in patients despite being known to induce premature epiphyseal closure [5–7].

One serious side effect of 13-cis-retinoic acid is irreversible premature epiphyseal closure in growing children [5]. The frequency of epiphyseal closure associated with systemic retinoid therapy is related to dose, age at exposure, and duration of treatment [8]. As the number of patients treated by 13-cis-retinoic acid increases, demands for salvage treatment after systemic retinoid therapy are emerging.

Recently, we encountered a case of bilateral knee varus deformity due to premature epiphyseal closure that occurred during treatment with isotretinoin for the treatment of neuroblastoma. To the best of our knowledge, this represents the first report of correction osteotomy of varus knee deformity occurring during treatment with isotretinoin.

## Case-presentation

A 10-year-old Japanese girl presented to our hospital with progressive pain in the right knee and varus deformity of bilateral knees. She had been diagnosed at three months old as having neuroblastoma from the right adrenal grand with multiple distant metastasis (bone marrow, liver, lung, thoracic wall and skin). Standardized protocol of the Study Group of Japan (A1 protocol [9], consisting of cyclophosphamide (1200 mg/m^2^), vincristine (1.5 mg/m^2^), tetrahydropyranyl adriamycin (pyrarubicin; 40 mg/m^2^), and cisplatin (90 mg/m^2^), was initiated after diagnosis. However, the chemotherapy was stopped due to multiple organ failure. As the next in line therapy 13-cis-retinoic acid was administrated. Administration of isotretinoin was subsequently initiated. She did not receive any other therapy in the last 10 years after administration of isotretinoin. The patient first complained of vague, transient pain in the right knee at 8 years old however she did not receive an additional check up by an orthopaedic surgeon.

The patient was of normal stature at presentation (height, 146 cm at 87th percentile; body weight, 37.6 kg at 62th percentile; body mass index, 17.6 kg/m^2^ at 62th percentile). Bilateral knees showed varus deformity, with worse deformity in the right knee than in the left knee (hip-knee-ankle angle [HKA]: right, − 23°; left, − 10°). The right knee joint showed a range of motion from 10° to 140° of flexion. Physical examination and radiographs showed a limb length discrepancy, with the right lower extremity 0.9 cm shorter than the left lower extremity (right femur, 38.1 cm; right tibia, 34.7 cm; right lower extremity, 72.8 cm; left femur, 38.3 cm; left tibia, 35.4 cm; left lower extremity, 73.7 cm). Radiographs and magnetic resonance imaging of the lower extremities revealed medial physeal arrests in both distal femoral physes and in both proximal tibial physes (Figs. 1a, 2) and additional abnormalities in bending of both femoral necks and shortage of both first metatarsals (Fig. 1b, c). According to image findings, a dysplasia epiphysealis multiples should be considered as differential diagnosis. However, the patient did not have family history for dysplasia epiphysealis and received systemic administration of 13-cis-retinoic acid for a long-time period. Considering all of these factors, we concluded that the growth disturbance in this patient was mainly due to premature epiphyseal closure caused by 13-cis-retinoic acid administration.

**Fig. 1:**
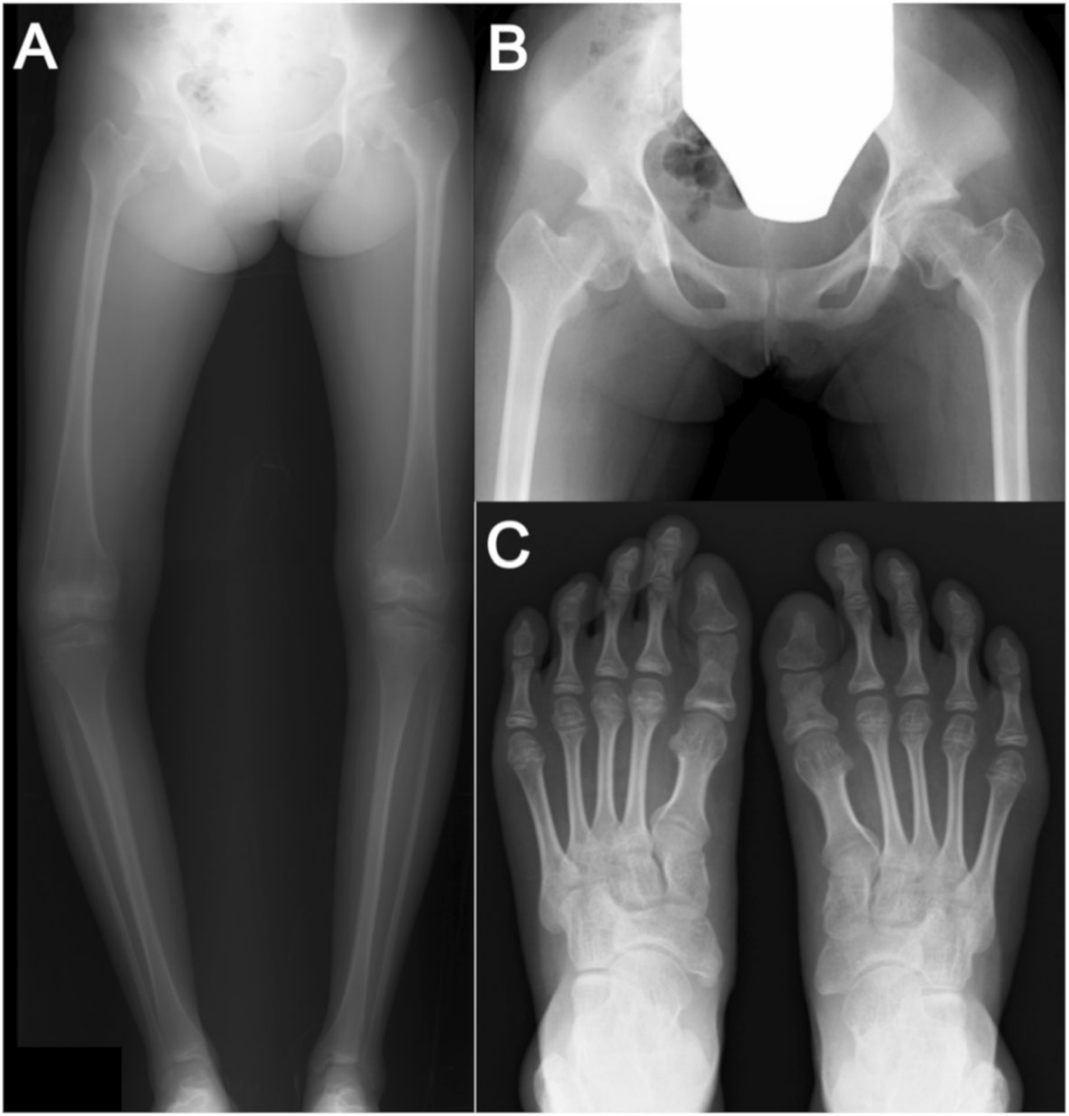
Abnormalities after long-term treatment for neuroblastoma using isotretinoin, a vitamin A derivative. **a** Medial physical arrests of both distal femoral physes and distal tibial physes. **b** Bending of both femoral necks and deformities of both femoral heads. **c** Both first metatarsal bones are short

**Fig. 2:**
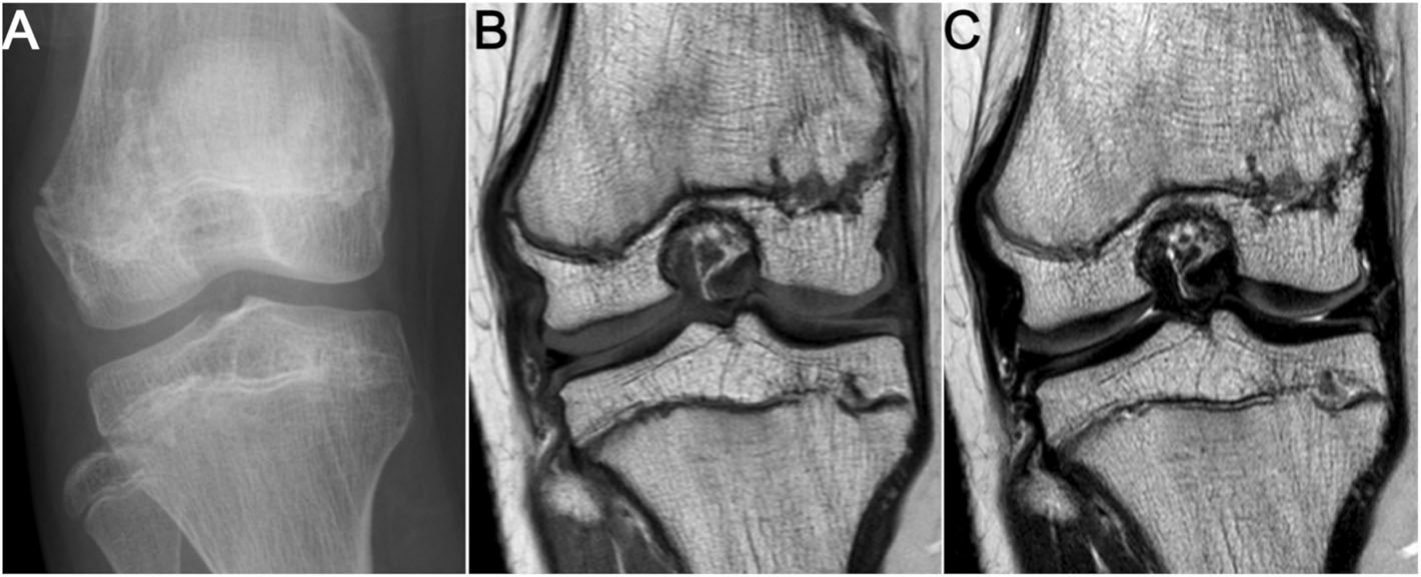
Close-up view of right distal femoral and proximal tibial physeal arrest. **a** Anteroposterior radiograph of right knee joint. **b**, **c** Coronal T1-weighted spin-echo (**b**) and T2-weighted spin-echo (**c**) MR images

Weight-bearing anteroposterior (AP) and lateral radiographs were taken preoperatively. The medial proximal tibial angle (MPTA) was 83° and the mechanical lateral distal femoral angle (mLDFA) was 107° with 1 cm leg discrepancy. Accordingly, we performed correction surgery using TSFs to correct both the varus deformity of distal femur and the leg discrepancy. Correction planning was performed using long leg standing AP radiographs. Preoperative planning was carried out using the online Spatial frame software package (http://www.spatialframe.com). Correction surgery was performed under general anesthesia with the patient supine. First, Taylor Spatial Frames (TSFs) were applied to the distal femur and an Ilizarov frame was fixated to the proximal tibia across the knee joint with tensioned wires and half pins. Second, osteotomy of the distal femur was performed. The angle of the osteotomy was aimed toward perpendicular against the mechanical axis of the lower extremity (Fig. 3a). After mounting the frame on the lower extremity, the mounting parameters were recorded and entered into TSF-dedicated software along with the deformity parameters. All corrections were made gradually after a latency phase of 7 days. The patient was instructed to perform gradual adjustments of the six struts of the TSF three times per day. Correction was continued until HKA had been corrected to 0° and a 1-cm elongation of the femur, which occurred 18 days after correction. After completing correction, the frame at the tibia was removed and the TSFs were connected with rods (Fig. 3b). Four months later, bone union was confirmed and the external fixator was removed (Fig. 3c). The MPTA was 85° and mLDFA was 86° without leg discrepancy (right femur, 38.9 cm; right tibia, 34.7 cm; right lower extremity, 73.9 cm; left femur, 38.4 cm; left tibia, 35.4 cm; left lower extremity, 74.2 cm). Arthroscopic investigation revealed that the articular surface of the right knee joint was intact (Fig. 3d). With the consent of the patient and her parents growth plate cartilage was arthroscopically harvested from the non-weight bearing area of the femoral intercondylar fossa to evaluate bone quality affected by 13-cis-retinoic acid administration. We minimized the impact on the growth plate, resulting that no further growth disturbance was observed in her right knee. Specimens (φ2.7 mm) were fixed in 10% buffered formalin and decalcified in 10% ethylenediaminetetraacetic acid. Each tissue sample was dehydrated, embedded in paraffin, and sectioned. To investigate chondrocyte apoptosis, the terminal deoxynucleotidyl transferase deoxyuridine triphosphate nick end labeling (TUNEL) assay was performed using an in situ Apoptosis Detection Kit following the instructions of the manufacturer (Takara, Japan). Histological findings revealed that the columnar structure of the growth plate had disappeared and cells were scattered (Fig. 4a). The stainability of Safranin-O staining in growth plate cartilage was dramatically reduced (Fig. 4b). In contrast, cells showing positive results for TUNEL staining were barely detectable (Fig. 4c). Postoperatively, the patient showed no pain in the right knee.

**Fig. 3:**
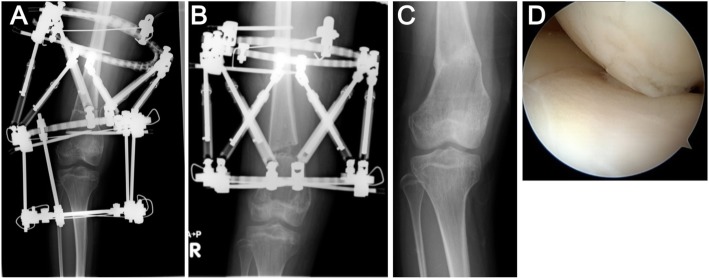
Postoperative anteroposterior radiographs and arthroscopic images of the patient by TSF. **a** Immediately after femoral osteotomy. **b** Gradual correction was achieved over the course of 18 days. **c** Four months later, bone union was confirmed and the external fixator was removed. **d** Arthroscopic findings showing an intact articular surface

**Fig. 4:**
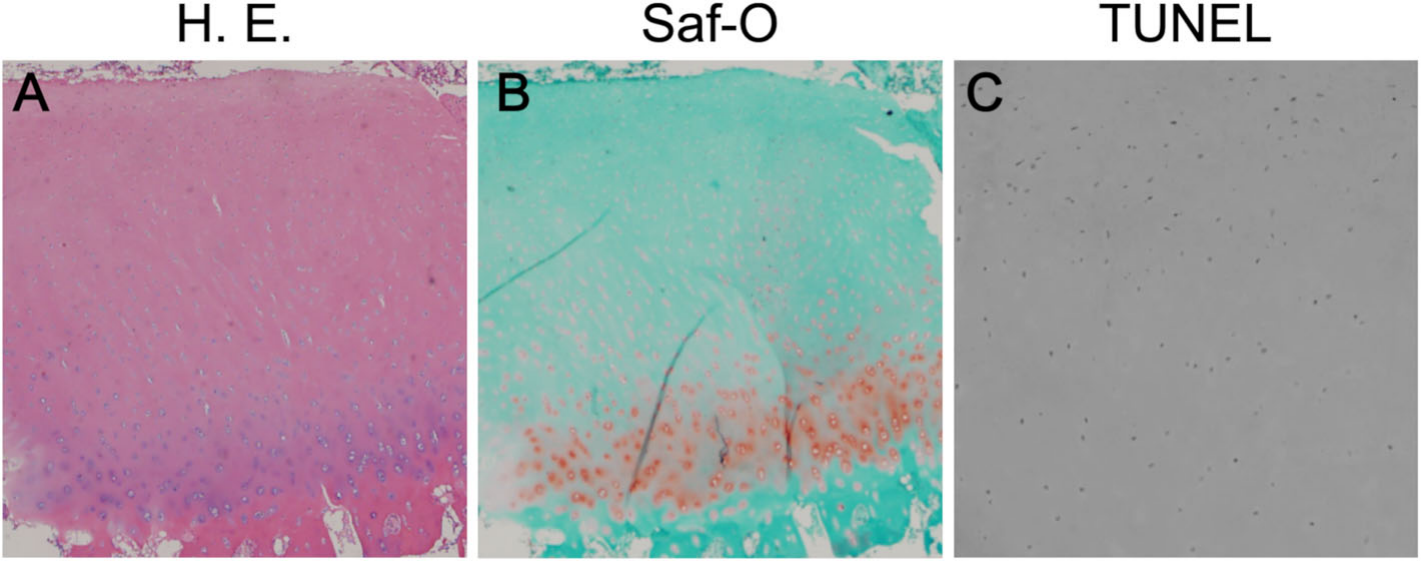
Histopathological findings of distal femoral growth plate cartilage. **a** Hematoxylin and eosin staining (HE) shows polar irregularity. **b** Safranin-O staining (Saf-O) shows decreased cartilage matrix. **c** TUNEL-stained section shows no obvious apoptosis in the growth plate cartilage. Original magnification: × 100 for A and B, × 400 for C

Eight years later, the patient presented again with progressive pain of the left knee joint and planned correction surgery for varus deformity of the left knee (Fig. 5a, b; HKA: − 9°). Mild tenderness was evident medially. The left knee joint had a range of motion from 0° to 145° flexion. MPTA was 88° and mLDFA was 103° without leg discrepancy (right femur, 40.1 cm; right tibia, 35.6 cm; right lower extremity, 75.7 cm; left femur, 40.0 cm; left tibia, 35.8 cm; and left lower extremity, 75.8 cm). Accordingly, we performed femoral closed wedge osteotomy because her epiphysis was fused and the center of the varus deformity was located in the distal femur, not in the tibia. Under general anesthesia, a longitudinal surgical incision measuring approximately 10 cm was made on the lateral aspect of the femur and the iliotibial band was split. The vastus lateralis muscle was then elevated anteriorly and the osteotomy site was exposed. Under fluoroscopic control, a Kirschner wire was inserted to the starting point for distal osteotomy at the lateral femoral epicondyle. Two Kirschner wires were then inserted for an oblique down-sloping wedge. A biplanar osteotomy was then completed and the wedge was gradually closed. A TomoFix Medial Distal Femur (Synthes, Solothurn, Switzerland) for the right knee was inserted on the lateral side of the femur (Fig. 5c). Partial weight-bearing was gradually permitted, with full weight-bearing permitted from postoperative week 6. Postoperative MPTA was 88° and mLDFA was 87°. The inserted plate was removed at 9 months postoperatively. No recurrence of varus deformity was evident on follow-up at 1 year (Fig. 5d). The patient remains able to go about her daily life without experiencing any knee pain.

**Fig. 5:**
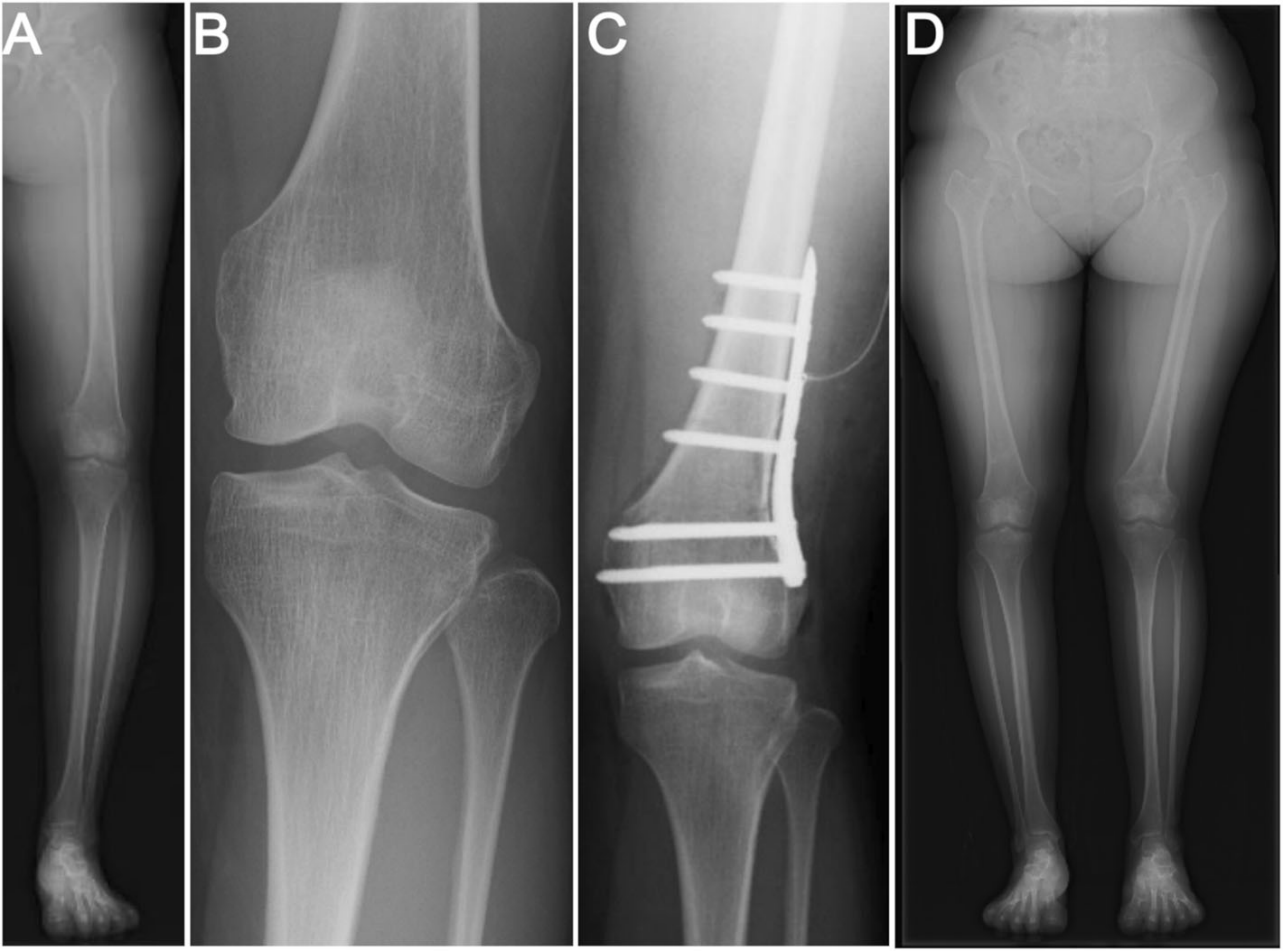
Pre- and postoperative anteroposterior radiographs of the patient by closed wedge osteotomy. **a**, **b** Left knee joint showing varus deformity. **c** Immediately after closed wedge osteotomy. **d** Final follow-up anteroposterior radiographs of the patient

## Discussion and conclusions

Premature epiphyseal closure caused by hypervitaminosis A can result in various outcomes, from transient abnormalities to permanent deformities. In a study with guinea pigs treated using various doses of vitamin A, the frequency of premature epiphyseal closure depended on the drug dose [10], suggesting that the dose of vitamin A might be important in determining the fate of the epiphyseal plate. In our case, oral isotretinoin was initially administered at 120 mg/day, and was still being administered at 40 mg/day on first admission, with treatment for a total period of 9.8 years from 1 year old. This dose of vitamin A arrested development of the femoral and tibial proximal growth plates, especially on the medial side, resulting in the patient developing severe knee varus deformity by 10 years old.

Retinoic acid, a major metabolite of vitamin A, is known to reduce cartilage matrix synthesis and enhance catabolism of cartilage proteoglycans in vitro [11–13]. These results suggest that the cartilage matrix in the growth plate might undergo histological changes in patients with hypervitaminosis A. In our case, 13-cis-retinoic acid accelerated the degradation of cartilage matrix in the growth plate, resulting in early closure of the growth plate. In contrast, apoptosis was not observed in the growth plate cartilage even though 13-cis-retinoic acid is known to induce cell death in tumors. Additionally, the arthroscopic findings showed that the articular surface was intact. These results suggested that hypervitaminosis A might affect the growth plate cartilage and induce epiphyseal closure without apoptosis.

The complications of osteoporosis and thinning of the long bones is known in hypervitaminosis A [14]. Severe osteoporosis and thinning of the long bones associated with this disease results in consistent occurrence of spontaneous bone fractures. However, few studies have described the surgical treatment of this disease. In our case, the right femoral bone revealed thinning and bone mineral density at L2-L4 did not show severe osteoporosis (0.962 g/cm^2^). We decided to extend the external fixation device below the knee only for the period of alignment correction as a safe option, and observed bone union as good as that seen in typical cases. As for the right knee joint, we performed femoral closed wedge osteotomy because her epiphysis was fused and the center of the varus deformity was located in the distal femur, not in the tibia. Bone union was achieved, as in the left knee joint.

This is the first report of arthroscopic and histological findings in premature epiphyseal closure caused by hypervitaminosis A. We believe that the clinician should not hesitate to administrate 13-cis-retinoic acid if the standard chemotherapy is not fully effective for neuroblastoma. In this paper, we reported about a patient who was successfully treated by correction surgery on bilateral premature epiphyseal closure due to systemic administration of 13-cis-retinoic acid. Treatment options for growth disturbance are available even after long term administration of 13-cis-retinoic acid. In addition, in cases like this a regular check of growth disturbance by a pediatric orthopaedic surgeon is required to insure a long term successful outcome.

## Data Availability

This is a case report of a single patient, to protect privacy and respect confidentiality; none of the raw data has been made available in any public repository. The original operation reports, intra-operative photographs, imaging studies and outpatient clinic records are retained as per normal procedure within the medical records of our institution. All data concerning the case are presented in the manuscript.

## Abbreviations

AP: Anteroposterior
HKA: Hip-knee-ankle angle
mLDFA: Mechanical lateral distal femoral angle
MPTA: Medial proximal tibial angle
TSFs: Taylor Spatial Frames
TUNEL: Terminal deoxynucleotidyl transferase deoxyuridine triphosphate nick end labeling
